# Mitochondrial pyruvate carrier 1: a novel prognostic biomarker that predicts favourable patient survival in cancer

**DOI:** 10.1186/s12935-021-01996-8

**Published:** 2021-05-31

**Authors:** Chen Xue, Ganglei Li, Zhengyi Bao, Ziyuan Zhou, Lanjuan Li

**Affiliations:** 1 State Key Laboratory for Diagnosis and Treatment of Infectious Diseases, National Clinical Research Center for Infectious Diseases, Collaborative Innovation Center for Diagnosis and Treatment of Infectious Diseases, The First Affiliated Hospital, College of Medicine, Zhejiang University, No. 79 Qingchun Road, Shangcheng District, Hangzhou, 310003 China; 2 Department of Neurosurgery, The First Affiliated Hospital, College of Medicine, Zhejiang University, Hangzhou, 310003 China

**Keywords:** MPC1, Metabolic reprogramming, Cancer, Glycolytic

## Abstract

Mitochondrial pyruvate carrier 1 (MPC1) is a key metabolic protein that regulates the transport of pyruvate into the mitochondrial inner membrane. MPC1 deficiency may cause metabolic reprogramming. However, whether and how MPC1 controls mitochondrial oxidative capacity in cancer are still relatively unknown. MPC1 deficiency was recently found to be strongly associated with various diseases and cancer hallmarks. We utilized online databases and uncovered that MPC1 expression is lower in many cancer tissues than in adjacent normal tissues. In addition, MPC1 expression was found to be substantially altered in five cancer types: breast-invasive carcinoma (BRCA), kidney renal clear cell carcinoma (KIRC), lung adenocarcinoma (LUAD), pancreatic adenocarcinoma (PAAD), and prostate adenocarcinoma (PRAD). However, in KIRC, LUAD, PAAD, and PRAD, high MPC1 expression is closely associated with favourable prognosis. Low MPC1 expression in BRCA is significantly associated with shorter overall survival time. MPC1 expression shows strong positive and negative correlations with immune cell infiltration in thymoma (THYM) and thyroid carcinoma (THCA). Furthermore, we have comprehensively summarized the current literature regarding the metabolic reprogramming effects of MPC1 in various cancers. As shown in the literature, MPC1 expression is significantly decreased in cancer tissue and associated with poor prognosis. We discuss the potential metabolism-altering effects of MPC1 in cancer, including decreased pyruvate transport ability; impaired pyruvate-driven oxidative phosphorylation (OXPHOS); and increased lactate production, glucose consumption, and glycolytic capacity, and the underlying mechanisms. These activities facilitate tumour progression, migration, and invasion. MPC1 is a novel cancer biomarker and potentially powerful therapeutic target for cancer diagnosis and treatment. Further studies aimed at slowing cancer progression are in progress.

## Background

Mitochondria are involved in bioenergetic, biosynthetic, and signalling organelle functions [1]. Mitochondria function as energy factories for cells and are essential for the activity, function, and viability of eukaryotic cells [2–4]. They are ubiquitous intracellular semiautonomous organelles responsible for bioenergetic metabolism, ageing, and apoptosis. Due to the high energy demand, mitochondria are especially important for oxidative phosphorylation (OXPHOS) [5, 6]. Metabolic reprogramming is a crucial hallmark of many diseases, including cancer. Metabolic adaptations are involved in tumour initiation and proliferation [7–9]. Mitochondrial dysfunction plays a crucial role in cancer metabolism, proliferation, and progression [10].

Metabolic alterations result in oncogenic metabolites in some malignancies [2, 11]. Metabolic reprogramming helps provide ATP and essential macromolecules for protein and nucleotide biosynthesis in cancer cells [12–15]. Cancer cells undergo metabolic reprogramming that enhances metabolic plasticity, enables the tumour to survive in a nutrient-scarce environment [16], and facilitates survival, proliferation, and metastasis [7, 17, 18]. Tumour cells typically display several, but not necessarily all, hallmarks of cancer, such as decreased glucose uptake, opportunistic nutrient acquisition, glycolysis, increased nitrogen assimilation, aberrant regulation of metabolically driven genes, and increased lactate production [7, 19, 20]. Aerobic glycolysis, also known as the Warburg effect [13], is one of the earliest altered metabolism phenotypes seen in tumour cells [21, 22]. Most cancers exhibit the Warburg effect while retaining mitochondrial respiration [23]. Aerobic glycolysis end products such as lactate may contribute to microenvironment alterations and facilitate tumorigenesis and cancer progression [22, 24, 25].

**Fig. 1:**
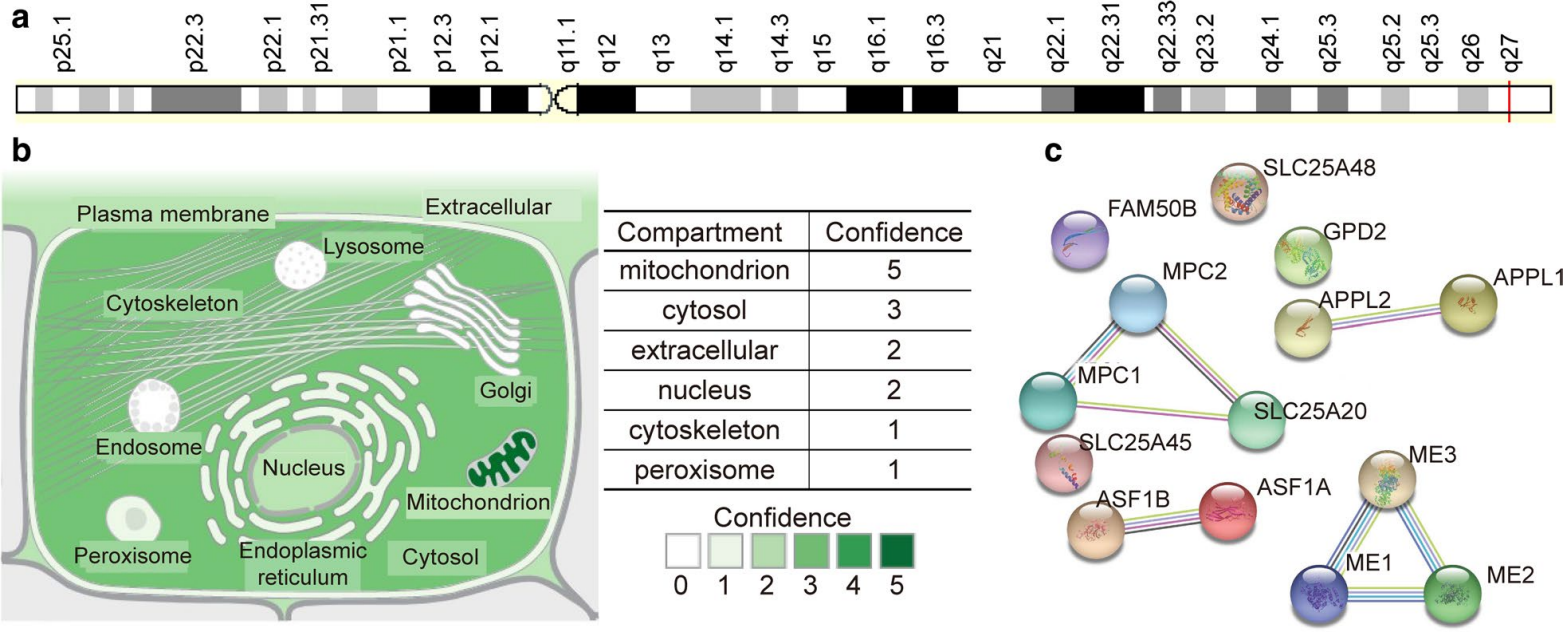
Basic information on MPC1. **a** The genomic location of the MPC1 gene. **b** The subcellular location of MPC1 (mainly mitochondrial). **c** Proteins that interact with MPC1

Alterations in mitochondria and mitochondrial DNA have been reported in many cancers [26–28]. Therefore, mitochondria may be an attractive therapeutic target for these cancers. Mitochondrial pyruvate carrier (MPC) is a conserved protein complex located in the mitochondrial membrane [29, 30]. The complex contains MPC1 and MPC2 proteins and plays a crucial function in transporting pyruvate, the end product of glycolysis, from the cytosol into mitochondria [31, 32]. Downregulation of MPC1 is associated with poor cancer prognosis, and MPC1 is particularly interesting as a potential therapeutic target. The MPC1 HUGO Gene Nomenclature Committee (HGNC) ID is 21606, and its genomic location is shown in Fig. 1a. MPC1 was distributed in mitochondria, the cytosol, the extracellular space, and the nucleus (Fig. 1b). The genomic information for MPC1 is presented in the form of a gene card (https://www.genecards.org/). Together, MPC1 and MPC2 function as crucial mitochondrial pyruvate transporters. MPC1 and MPC2 form a protein complex in the inner mitochondrial membrane [33]. Importantly, the MPC1 pyruvate transporter acts as a bridge between glycolysis and the tricarboxylic acid (TCA) cycle. The loss of MPC1 in humans may result in impaired mitochondrial pyruvate uptake and pyruvate oxidation [33]. Pathway enrichment analyses have indicated that MPC1 interacts with many proteins. The top 13 proteins that interact with MPC1 are shown in Fig. 1c. Some biological processes regulated by MPC1 are glucose energy metabolism, pyruvate metabolism and the TCA cycle, respiratory electron transport, ATP synthesis via chemiosmotic coupling, heat production via uncoupling proteins, and glucose metabolism.

Overexpression of MPC1 promotes metabolic flux through the mitochondrial membrane and inhibits the Warburg effect without compromising glucose consumption or maximum cell concentration [34]. In contrast, MPC1 deficiency, glutamine breakdown, increased urea, and increased pyruvate-alanine cycle activity all lead to better regulation of gluconeogenesis and maintenance of euglycaemia [35]. A high-fat diet in mice may increase hepatic MPC1 expression and activity [35], which contribute to gluconeogenesis and hyperglycaemia [36]. MPC1 may function in fatty acid oxidation to meet biological energy demands [35]. The overexpression of MPC1 significantly reduces lactate production [34].

## Decreased MPC1 expression may function in tumour progression

MPC1 dysregulation is associated with cancer. Notably, Bensard et al. [9] demonstrated that MPC1 has a close relationship with the glycolytic metabolic phenotype and stem cell markers in the cancer initiation process. MPC1 is downregulated in various cancers. Aberrant expression of MPC1 is also involved in cancer-associated metabolic dysregulation. For example, MPC1 regulates mitochondrial respiratory capacity in renal cell carcinoma (RCC) [37], prostate cancer [38], hepatocellular carcinoma (HCC) [39], and cholangiocarcinoma [40].

**Fig. 2:**
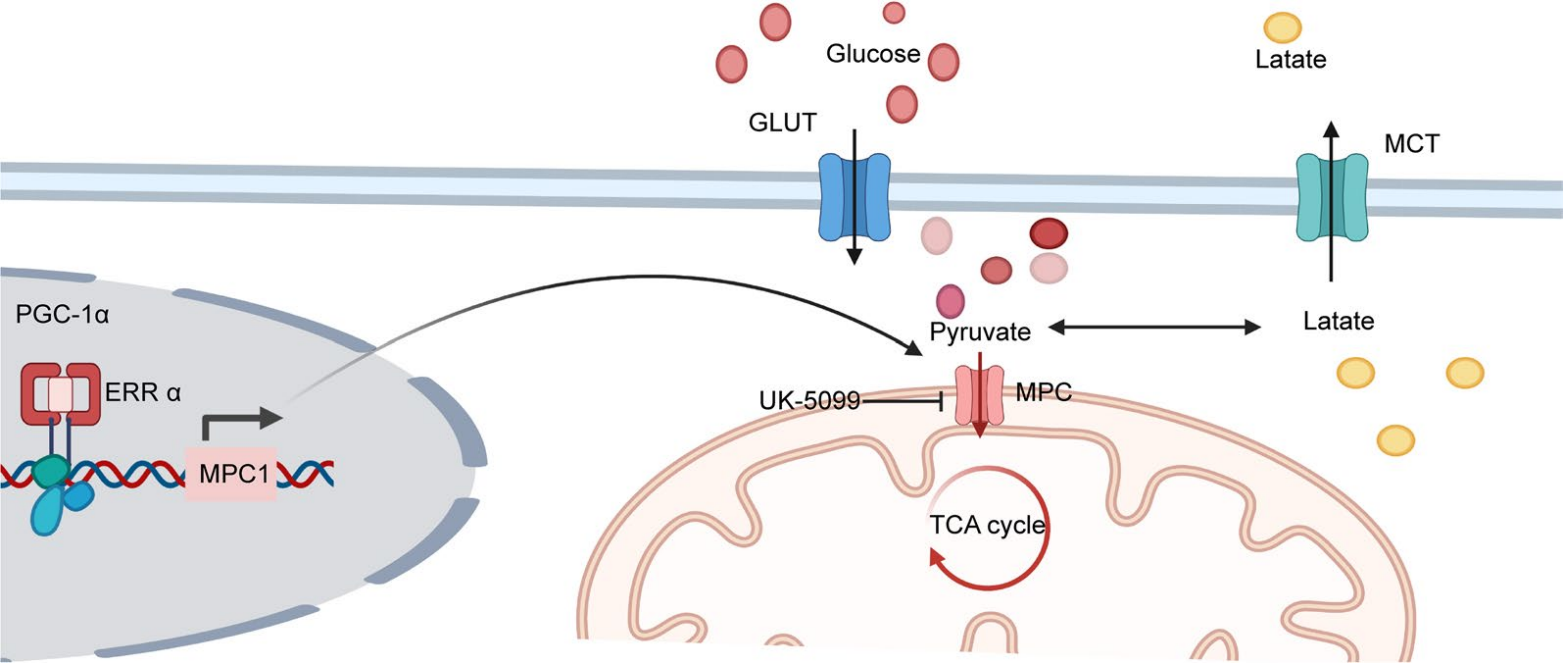
The function of MPC1 in mitochondria. Oestrogen-related receptor-alpha (ERR-α) activates the proximal MPC1 promoter through PGC-1α, thus causing MPC1 overexpression. PGC-1α promotes MPC1 expression, enhancing pyruvate transport and OXPHOS activity

**Fig. 3:**
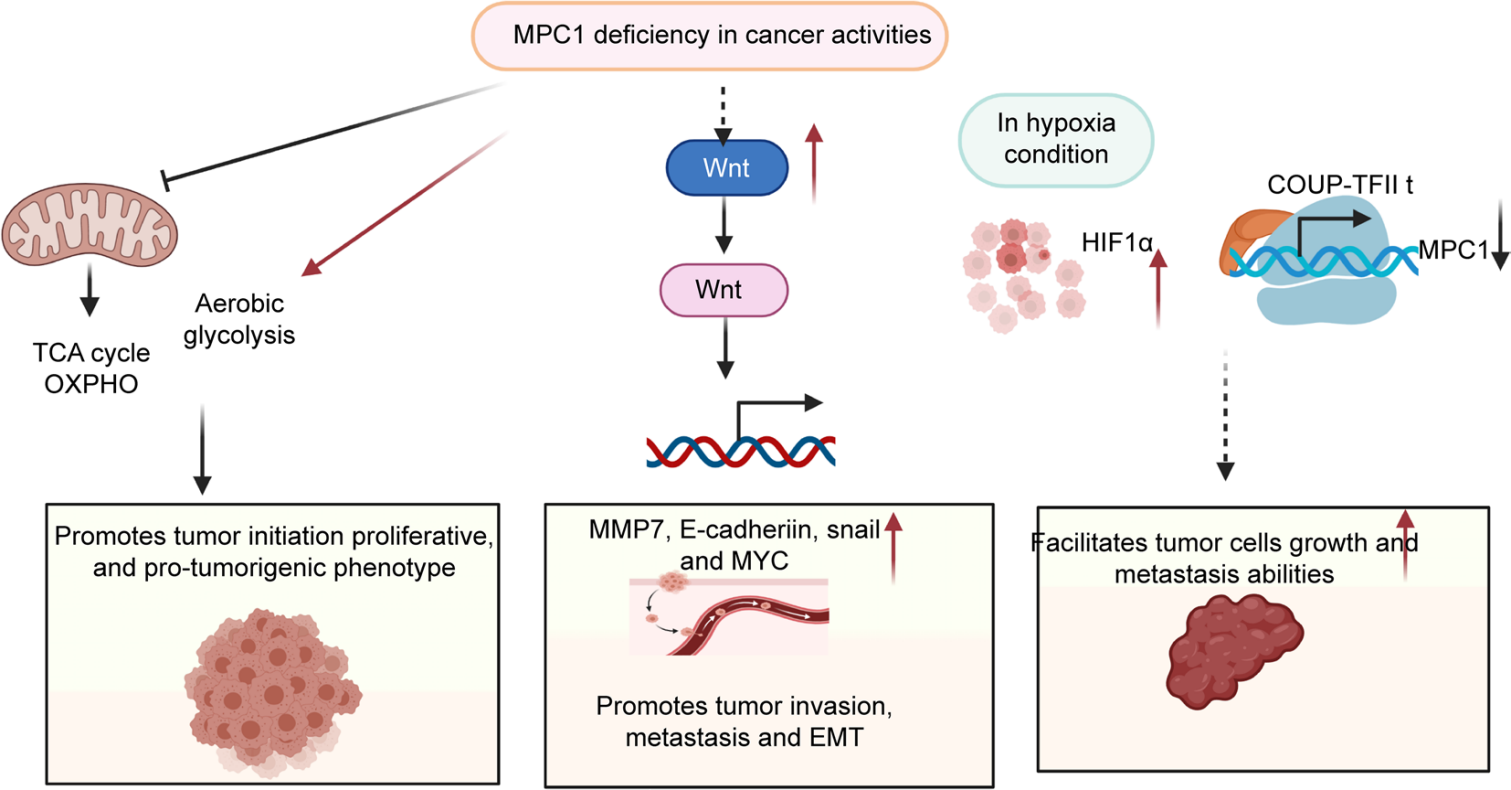
MPC1 deficiency facilitates tumour progression, migration, and invasion

MPC1 is downregulated in many tumour cells, which affects tumour mitochondrial respiratory capacity. In human renal cell carcinoma, the upstream regulator peroxisome proliferator-activated receptor-gamma coactivator 1 alpha (PGC-1α) targets and recruits oestrogen-related receptor-alpha (ERR-α). The accumulation of ERR-α activates the proximal MPC1 promoter, thus causing MPC1 overexpression. In addition, PGC-1α deficiency inhibits MPC1 expression, therefore decreasing pyruvate transport and impairing pyruvate-driven OXPHOS in RCC [41] (Fig. 2). PGC-1α is downregulated in HCC, leading to decreased MPC1 expression. In the liver, MPC1 binds nuclear respiratory factor 1 (NRF1) and promotes mitochondrial biogenesis. In HCC, a decrease in proliferator-activated receptor gamma coactivator-1 alpha(PGC1α)attenuates NRF1 and MPC1 expression, thus promoting HCC progression [39]. In prostate cancer, MPC1 deficiency significantly increases lactate production, glucose consumption, and glycolytic capacity [38]. In cholangiocarcinoma, PGC1α reverses the Warburg effect by switching Warburg effect-related aerobic glycolysis to OXPHOS and upregulating MPC1 and pyruvate dehydrogenase E1α subunit expression [40], which facilitate tumour migration and invasion (Figs. 2, 3).

## MPC1 functions as a clinical indicator of many cancers

### Correlations of MPC1 expression with infiltrating immune cells across cancers

**Fig. 4:**
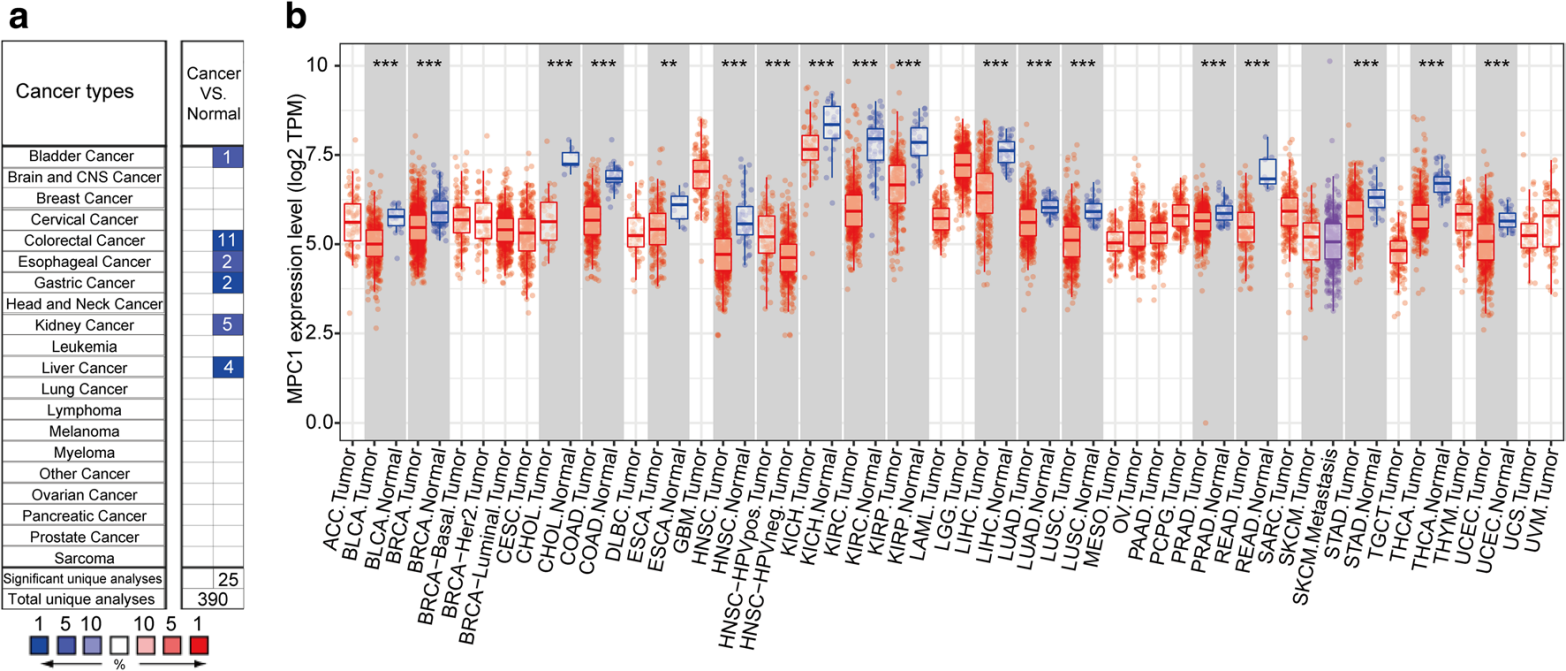
MPC1 is differentially expressed in various cancers. **a** Comparison of MPC1 expression levels in cancer tissues and normal tissues via the Oncomine database. **b** The mRNA expression level in different tumours according to the TIMER2.0 analysis of TCGA data (*P < 0.05, **P < 0.01, ***P < 0.001)

**Fig. 5:**
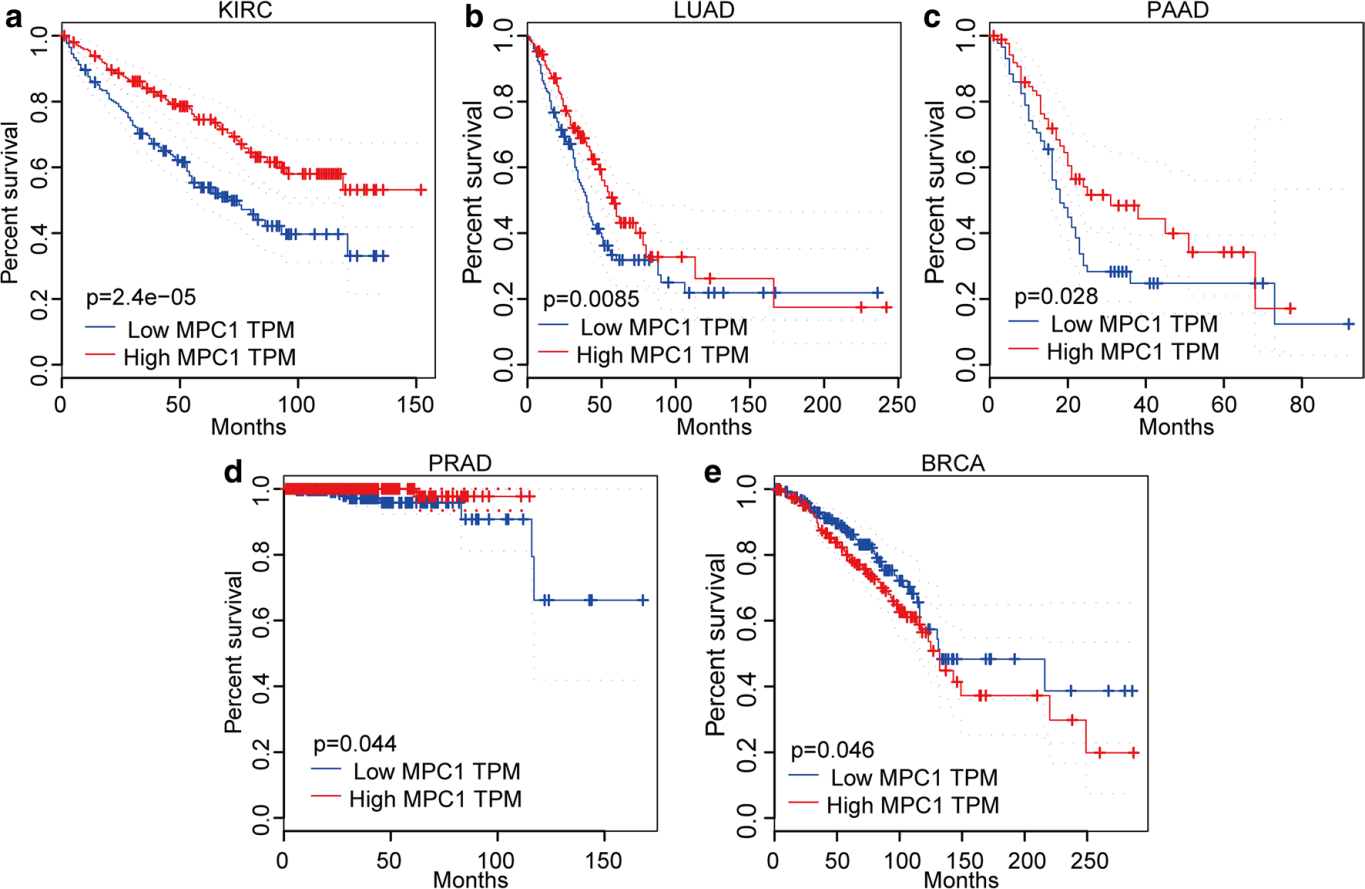
Prognostic analysis of high and low MPC1 expression in various cancers. **a** Survival curves of OS in the KIRC cohort. **b** Survival curves of OS in the LUAD cohort. **c** Survival curves of OS in the PAAD cohort. **d** Survival curves of OS in the PRAD cohort. **e** Survival curves of OS in the BRCA cohort

To explore the mRNA expression level in tumours and adjacent normal tissues, we utilized the Oncomine database (https://www.oncomine.org/). The results implied that MPC1 is differentially expressed in various cancers; for example, MPC1 is expressed at lower levels in bladder, colorectal, oesophageal, gastric, kidney, and liver cancers than in adjacent normal tissues (Fig. 4a). To investigate the MPC1 expression level in The Cancer Genome Atlas (TCGA) database, TMIER 2.0 online analysis was employed in this study. The investigation demonstrated that MPC1 was lower in bladder urothelial carcinoma (BLCA), breast invasive carcinoma (BRCA), cervical squamous cell carcinoma and endocervical adenocarcinoma (CESC), cholangiocarcinoma (CHOL), colon adenocarcinoma (COAD), oesophageal carcinoma (ESCA), head and neck squamous cell carcinoma (HNSC), kidney chromophobe (KICH), kidney renal clear cell carcinoma (KIRC), kidney renal papillary cell carcinoma (KIRP), liver hepatocellular carcinoma (LIHC), lung adenocarcinoma (LUAD), lung squamous cell carcinoma (LUSC), prostate adenocarcinoma (PRAD), rectum adenocarcinoma (READ), stomach adenocarcinoma (STAD), thyroid carcinoma (THCA), and uterine corpus endometrial carcinoma (UCEC) than in adjacent normal tissues (Fig. 4b). Furthermore, we explored the correlation between MPC1 expression and patient prognosis. The results indicated that MPC1 was significantly differentially expressed in many cancers, such as BRCA, KIRC, LUAD, pancreatic adenocarcinoma (PAAD), and PRAD. However, in KIRC (P = 2.4e-05, Fig. 5a), LUAD (P = 0.0085, Fig. 5b), PAAD (P = 0.028, Fig. 5c), and PRAD (P = 0.044, Fig. 5d), high expression of MPC1 was closely associated with favourable prognosis. Low expression of MPC1 in BRCA was significantly associated with shorter overall survival time (P = 0.046, Fig. 5e). In addition, MPC1 expression was deeply affected in five cancer types: BRCA, KIRC, LUAD, PAAD, and PRAD. However, in KIRC, LUAD, PAAD, and PRAD, high expression of MPC1 was closely associated with favourable prognosis. Because our aim was to determine the expression patterns and prognostic value of MPC1 across cancers, we analysed the expression status of MPC1 at the mRNA level and then explored its relationship with prognosis across cancers using the TCGA database. MPC1 showed significantly different expression levels across cancers. Differential expression of MPC1 indicated different prognoses in various cancers. Based on an analysis of public data, the MPC1 mRNA expression level is a potential prognostic indicator of cancer. However, because of tumour heterogeneity, patient prognoses may be different. These studies imply that aberrant MPC1 expression has the potential to become a novel detection biomarker and prognostic indicator in various cancers.

#### MPC1 functions in T cell homeostasis

**Fig. 6:**
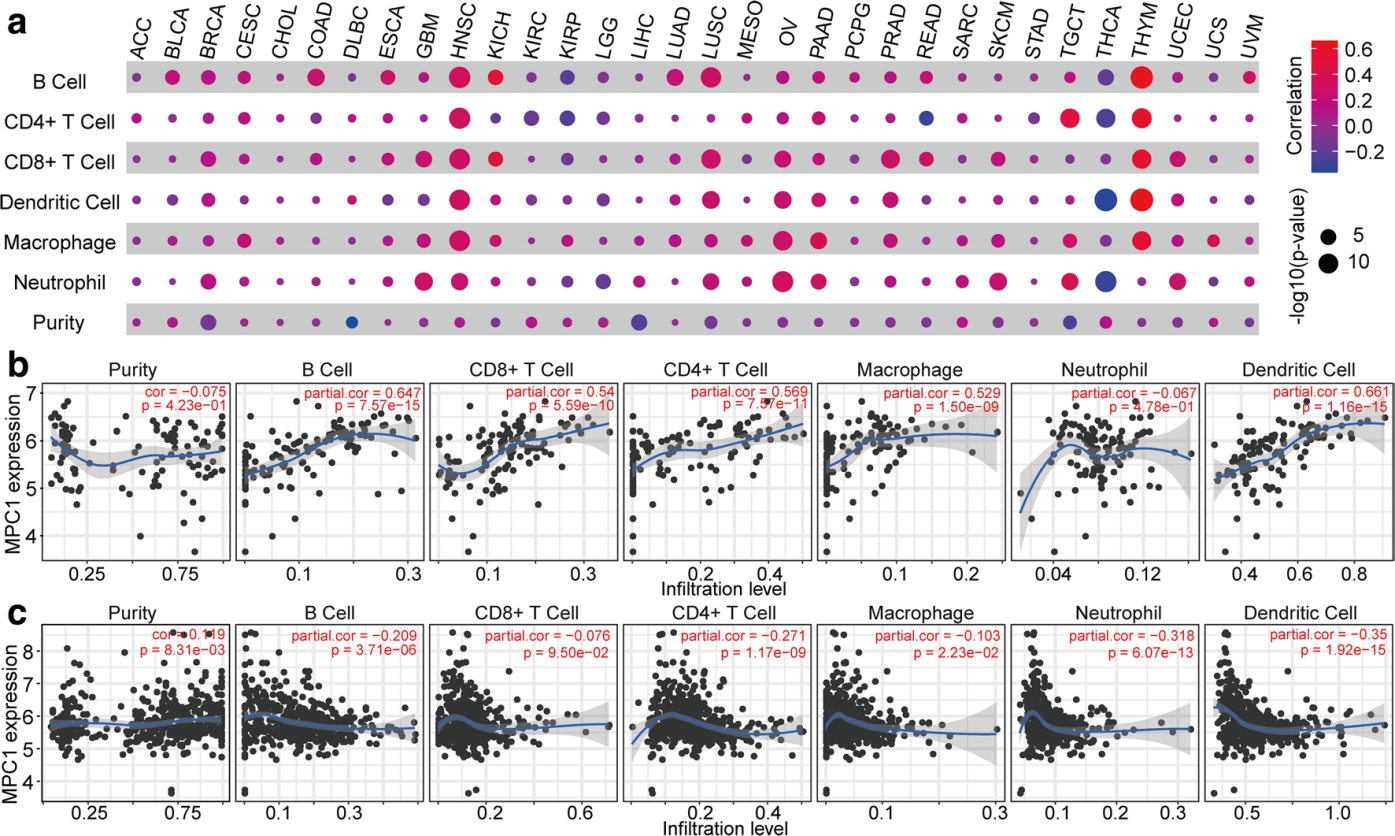
The association of MPC1 expression with immune infiltration. **a** MPC1 expression and the immune infiltration landscape in 26 cancers. **b** MPC1 expression is positively associated with tumour purity and immune cell infiltration in THYM. **c** MPC1 expression is negatively associated with tumour purity and immune cell infiltration in THCA

To estimate tumour-associated immune cell infiltration and MPC1 expression across cancers, we performed TIMER2.0 online analysis (http://timer.comp-genomics.org/), and the results demonstrated that MPC1 expression showed a significant relationship with immune purity and immune cell infiltration in 26 cancers, as illustrated in Fig. 6a. Specifically, B cell, CD8+ T cell, dendritic cell (DC), macrophage, and neutrophil infiltration showed a significant association with MPC1 expression across cancers. In addition, in HNSC, LUSC, ovarian cancer (OV), thymoma (THYM), THCA, and tenosynovial giant cell tumour (TGCT), there were strong correlations between the immune cell infiltration level and MPC1 expression. In addition, we further investigated the association of MPC1 expression with immune cell infiltration levels in the cancer types with the strongest positive and negative correlations and determined that MPC1 expression showed strong positive correlations with the levels of infiltrating B cells (r = 0.647, P = 7.57e-15), CD8+ T cells (r = 0.54, P = 5.59e-10), CD4+ T cells (r = 0.569, P = 7.57e-11), macrophages (r = 0.529, P = 1.50e-09), neutrophils (r = −0.067, P = 4.78e-01) and DCs (r = 0.661, P = 1.16e-15) in THYM (Fig. 6b). Similarly, there were negative correlations with immune purity (r = −0.149, P = 8.31e-03) and the levels of infiltrating B cells (r = −0.209, P = 3.71e-06), CD8+ T cells (r = −0.076, P = 9.50e-02), CD4+ T cells (r = −0.271, P = 1.17 e-09), macrophages (r = −0.103, P = 2.23e-02), neutrophils (r = −0.318, P = 6.07e-13), and DCs (r = −0.35, P = 1.92e-15) in THCA (Fig. 6c). These results indicate that MPC1 plays an important role in immune infiltration in various cancers.

## MPC1 function as a key indicator of many cancers in the clinic

Metabolic reprogramming is considered a hallmark of cancer and leads to tumour development [42]. Major metabolic aberration is associated with the metabolic environment of cancer [43]. Alternations in cancer metabolism further interact with cellular signalling and epigenetics to promote carcinogenesis and tumour development [44, 45]. MPC1 also functions as a clinical indicator of cancers, including lung cancer [46], gastric cancer [47], colorectal carcinoma (CRC) [9], intrahepatic cholangiocarcinoma (ICC) [48], RCC [49], and glioblastoma (GBM) [41]. In lung cancer, MPC1 expression is lower in LUAD tissue than in non-tumour tissue and is remarkably associated with favourable prognosis [46]. In prostate cancer, MPC1 expression is significantly decreased; this decrease is closely associated with unfavourable prognosis [38]. Similarly, Zhou et al. [47] found that MPC1 expression is decreased in gastric cancer tumour tissues compared with non-tumour tissues and that lower MPC1 expression predicts poor prognosis. Lower MPC1 expression is associated with advanced tumour stage, greater invasion depth, and lymph node metastasis [47]. Bensard et al. [9] found that MPC1 and MPC2 are decreased in human COAD, a result that is recapitulated in early-stage adenomas. Schell et al. [50] demonstrated that MPC1 has low expression in various cancers; they also verified that low expression of MPC1 is closely associated with cancer onset and poor prognosis in colon cancer. They used xenografts to validate that reintroduction of appropriate MPC1 expression reduces tumour growth. Similarly, Tian et al. [51] explored the TCGA and Gene Expression Omnibus databases and confirmed that MPC1 is downregulated in CRC compared with adjacent normal tissues. In addition, they also discovered that MPC1 expression gradually decreased from normal tissues to primary CRC tissues to CRC metastasis tissues [51]. Sandoval et al. [52] utilized TCGA data and found that MPC1 has low expression in COAD tumours with adenomatous polyposis coli (APC) deletions. APC is a tumour suppressor and dictates intestinal cell differentiation fate by regulating the pyruvate metabolism process. In ICC tissues, the expression of MPC1 as measured by immunohistochemistry is lower than that in non-tumour tissues, a finding that is closely associated with poor prognosis [48]. In RCC, Tang et al. [49] measured MPC1 expression in 10 pairs of RCC and corresponding adjacent non-cancerous tissues by qPCR and western blotting. The results indicated that MPC1 expression was significantly decreased in tumour tissues compared to that in non-cancerous tissues. Chai et al. [41] demonstrated that MPC1 is significantly downregulated in GBM tumour tissues, which was associated with poor prognosis, including poor response to temozolomide, based on TCGA database analysis.

## Decreased MPC1 expression promotes tumour progression

Cellular metabolism provides a permissive environment that generates metabolic intermediates regulating cellular growth [53–55] and proliferation [9]. Cancer cell invasion of adjacent tissues and metastasis to distant sites are complex [56], multiscale processes. Invasion and metastasis contribute to high recurrence risk, poor prognosis, and low survival [57, 58]. Cancer metabolism contributes to cancer cell viability and growth [59]. MPC1 deletion promotes tumour initiation as well as a proliferative and protumorigenic phenotype in genetic tumour models [9]. Loss of MPC1 blocks mitochondrial pyruvate oxidation, which facilitates aerobic glycolysis in colon cancer cells [9]. Ohashi et al. [48] found that MPC1 induces epithelial-to-mesenchymal transition in human ICC cell lines. In addition, low MPC1 expression is related to the invasion and metastasis of ICC cell lines.

MPC1 promotes metastatic capacity through metabolic reprogramming. MPC1 is significantly depressed in CRC [51], both in human tumour tissues and in mouse models. The MPC1 expression level in metastatic CRC is lower than that in primary CRC [51]. Decreased MPC1 expression promotes β-catenin nuclear transcription, enhances the Wnt/β-catenin pathway, and facilitates the expression of the cancer metastasis-related proteins MMP7, E-cadherin, Snail, and MYC, thus promoting CRC liver metastasis. Decreased MPC1 expression enhances lung cancer cell invasion and migration abilities via matrix metalloproteinase (MMP) pathways, and MMP2, MMP3, and MMP7 are significantly overexpressed in an MPC1 knockdown mouse model [46]. Invadopodia formation in MPC1-deficient groups is remarkably increased [46]. In lung cancer, MPC1 works with mitochondrial signal transducer and activator of transcription 3 (mito-STAT3) to reduce cytoplasmic STAT3. Inhibiting STAT3 phosphorylation attenuates malignant progression in lung cancer cells [46]. In mouse xenograft tumours, the overexpression of MPC1 reduces the tumour growth rate and tumour size [46]. MPC1 expression is significantly decreased in prostate cancer specimens [38]. Overexpression of MPC1 significantly inhibits the invasion ability of prostate tumour cells. Chicken ovalbumin upstream promoter transcription factor II (COUP-TFII), an MPC1 upstream regulator, represses MPC1 expression and facilitates growth and metastasis of prostate cancer cells [38]. MPC1 is also decreased in glioblastoma cell lines [60]. In an in vitro RCC experiment, MPC1 deficiency markedly increased the malignancy of RCC. MPC1 functions as an upstream regulator of hypoxia-inducible factor 1-alpha (HIF1α) and plays a key role in the hypoxia/HIF1α axis in oxygen-deficiency conditions. Decreased MPC1 expression facilitates the mobility and invasion of RCC cells through upregulation of MMPs [49]. Consistent with previous studies, the average weight and volume of RCC xenograft tumours in the MPC1-deficient groups were significantly greater than those in the MPC1 overexpression groups [49] (Fig. 3).

## The post-translational regulation of MPC1 in cancer

Metabolic and epigenomic alterations facilitate cancer development and progression [61, 62]. Metabolic remodelling and changes to the epigenome (including acetylation) have an important bidirectional regulatory mechanism in cancer [21, 63, 64]. Upon pyruvate transportation, sirtuin 3 (SIRT3) binds and stabilizes MPC1 through deacetylation, which enhances mitochondrial inner member transport of pyruvate [65] (Fig. 2). MPC1 is acetylated at the K45 and K46 acetylation sites. Under high glucose conditions, increased SIRT3-MPC1 binding and MPC1 deacetylation inhibit colon cancer cell growth [65]. MPC1 also plays an important role in enhancing cholangiocarcinoma cell migration and invasion capabilities [47]. In gastric cancer cell lines, the overexpression of MPC1 may attenuate proliferation, migration, and invasion. Taken together, these findings indicate that MPC1 is involved in various tumour progression processes and might be a promising target for cancer treatment.

## MPC1 and the stemness phenotype

The overexpression of MPC1 in CRC significantly inhibits the stemness and proliferation abilities of tumour suppressor-deficient intestinal stem cells [9]. LUAD cell lines with lentiviral vector-mediated MPC1 overexpression exhibited smaller volumes and numbers of tumour spheres. Decreasing the expression of MPC1, like MPC1 knockdown and MPC1 silencing, resulted in significantly increased tumour size and volume [46]. In addition, MPC1 deficiency significantly increased cancer stem cell markers such as Nanog homeobox (NANOG), octamer-binding transcription factor 4 (OCT4), and SRY-box transcription factor 2 (SOX2) in LUAD cells in vivo [46]. In gastric cancer, the overexpression of MPC1 decreases the stem cell-like properties and sphere formation capability of tumour cells, and stemness markers such as NANOG, OCT4, SOX2, and β-actin are significantly decreased [47]. In colon cancer cells, MPC1 deficiency promotes stem cell-like gene expression, whereas MPC1 overexpression inhibits stemness ability [9]. Schell et al. [6, 50] found that rescuing the expression of MPC enhances mitochondrial pyruvate oxidation. Specifically, increased MPC1 significantly impedes colony formation in soft agar, spheroid formation, and xenograft growth.

## MPC1 mediates metabolic processes

Metabolic substrates are essential for multiple pathological and physiological regulatory processes [31, 66, 67]. The targeting of a large number of molecules and regulatory pathways to slow disease progression, especially in cancers and Parkinson's disease, is under investigation. Studies have suggested MPC as a novel therapeutic target in Parkinson's disease progression [31]. A mechanism involving the MPC complex was found to be involved in enhancing autophagy via mammalian target of rapamycin (mTOR) activation and inhibiting neuroinflammation [31]. Aberrant expression of the MPC complex contributes to pyruvate transport abnormalities and is significantly correlated with cancer cell energy production, also called the Warburg effect [13, 68]. In the early stages of CRC, the glycolytic metabolic phenotype can be detected, featuring low expression of MPC1 [9]. In human renal cell carcinoma, decreased MPC1 expression might lead to impaired mitochondrial respiratory capacity in renal cell carcinoma cells through the upstream gene regulation of PGC1α [37]. These studies suggest that MPC1 is essential for chronic disease and cancer-associated phenotypes and functions as a master regulator of disease progression.

## MPC1 is involved in thermoregulation

MPC1 is involved in brown adipose tissue (BAT)-mediated energy metabolism. BAT transforms chemical energy into heat, which helps maintain a warm body temperature and regulates energy metabolism. MPC1 deficiency in BAT increases ketogenesis, causing an increase in 3-hydroxybutyrate in the blood under cold conditions. A significant decrease in MPC1 and MCP2 expression occurs in the BAT of male high-fat-diet mice [69].

## MPC1 functions in T cell homeostasis

In T cell homeostasis, MPC1 deficiency affects multiple pathways in early thymocyte T cell β-selection and positive selection [70]. MPC1 deficiency results in a significant decrease in specific αβ T cell numbers, and both reduces and activates peripheral T cell populations [70]. The pyruvate oxidation of T cell precursors is a crucial energy metabolic process for optimal αβ T cell maturation in thymic development [70].

## MPC1 is associated with drug resistance

Cancers with strong resistance to current treatments have a poor prognosis. Chai et al. [41] demonstrated the involvement of MPC1 in temozolomide drug resistance, which is closely related with poor prognosis. Oka et al. [71] showed that the relapse of patients with multiple myeloma treated with bortezomib is strongly associated with decreased MPC-1 expression. In addition, Kuroda et al.[72] classified myeloma cells according to phenotype and found that intermediate myeloma cells (MPC1+CD49e− CD45−) were remarkably suppressed. They also detected a large number of residual immature myeloma cells (MPC1− CD49e− CD45−/+) after 3 or 4 cycles of vincristine, doxorubicin, and dexamethasone chemotherapy [72]. However, the need for clinical MPC1-targeting therapies remains urgent.

## Conclusions

MPC1, located in the mitochondrial inner membrane, functions in transporting pyruvate from the cytoplasm into mitochondria [29]. To sustain tumour growth, cancer cells need to adapt to the acidic tumour microenvironment [42, 73, 74]. Decreased MPC1 expression facilitates a reduction in the conversion of pyruvate into circulating lactate and plays an important role in regulating cancer-associated metabolism. Specifically, the inhibition of MPC1 may attenuate pyruvate transport into the mitochondrial inner membrane. OXPHOS activity is thus significantly inhibited, and lactate production, glucose consumption, and glycolytic capacity are strongly enhanced, supporting the tumour microenvironment [75–78]. Moreover, MPC1 deficiency may contribute to the growth, invasion, and metastasis of cancer cells.

Targeting MPC1 may provide novel insight into the design and assessment of drugs for treating cancers, which may be a promising cancer treatment strategy for patients.

## Data Availability

The datasets used and/or analysed during the current study are available from the corresponding author upon reasonable request.

## Abbreviations

MPC1: Mitochondrial pyruvate carrier 1
OXPHOS: Oxidative phosphorylation
MPC: Mitochondrial pyruvate carrier
TCA: Tricarboxylic acid
HCC: Hepatocellular carcinoma
PGC-1α: Peroxisome proliferator-activated receptor-gamma coactivator 1 alpha
ERR-α: Oestrogen-related receptor-alpha
NRF1: Nuclear respiratory factor 1
BLCA: Bladder urothelial carcinoma
BRCA: Breast-invasive carcinoma
CESC: Cervical squamous cell carcinoma and endocervical adenocarcinoma
CHOL: Cholangiocarcinoma
COAD: Colon adenocarcinoma
ESCA: Oesophageal carcinoma
HNSC: Head and neck squamous cell carcinoma
KICH: Kidney chromophobe
KIRC: Kidney renal clear cell carcinoma
KIRP: Kidney renal papillary cell carcinoma
LIHC: Liver hepatocellular carcinoma
LUAD: Lung adenocarcinoma
LUSC: Lung squamous cell carcinoma
PRAD: Prostate adenocarcinoma
READ: Rectum adenocarcinoma
STAD: Stomach adenocarcinoma
THCA: Thyroid carcinoma
UCEC: Uterine corpus endometrial carcinoma
OV: Ovarian cancer
THYM: Thymoma
CRC: Colorectal carcinoma
ICC: Intrahepatic cholangiocarcinoma
RCC: Renal cell carcinoma
GBM: Glioblastoma
TCGA: The Cancer Genome Atlas
APC: Adenomatous polyposis coli
MMP: Matrix metalloproteinase
mito-STAT3: Mitochondrial signal transducer and activator of transcription 3
COUP-TFII: Chicken ovalbumin upstream promoter transcription factor II
HIF1α: Hypoxia-inducible factor 1-alpha
SIRT3: Sirtuin 3
BAT: Brown adipose tissue
NANOG: NANOG homeobox
SOX2: SRY-box transcription factor 2
OCT4: Octamer-binding transcription factor 4
mTOR: Mammalian target of rapamycin
